# Patient‐specific versus off‐the‐shelf unicompartmental knee arthroplasty during level walking

**DOI:** 10.1002/jeo2.70347

**Published:** 2025-08-27

**Authors:** Haithem M'barki, Etienne L. Belzile, Katia Turcot

**Affiliations:** ^1^ Department of Kinesiology, Faculty of Medicine Laval University Quebec City Quebec Canada; ^2^ Center for Interdisciplinary Research in Rehabilitation and Social Integration (Cirris) Quebec City Quebec Canada; ^3^ Department of Surgery, Division of Orthopedic Surgery CHU de Québec‐Université Laval Quebec City Quebec Canada

**Keywords:** gait analysis, kinetics, knee, patient‐specific implant, total support moment, unicompartmental knee arthroplasty

## Abstract

**Purpose:**

Personalised unicompartmental knee arthroplasty (UKA) improves tibial implant positioning and clinical outcomes compared to an off‐the‐shelf UKA. However, no biomechanical study has confirmed the functional superiority of custom implants. The study aimed to assess potential differences between personalised and off‐the‐shelf UKA in knee joint function in terms of both biomechanical and clinical outcomes during level walking.

**Methods:**

Twenty‐two patients and 22 healthy individuals (control group [CG]), matched for age and height, were recruited. Eight patients were implanted with a Bodycad UKS (BUKS) prosthesis, and 14 patients with an Oxford UKA (OUKA) prosthesis. Participants walked barefoot along a 10 m walkway. To quantify 3D kinematics and kinetics, a 10‐camera motion analysis system and four force plates were used. The knee injury and osteoarthritis outcome score (KOOS) was utilised to measure knee function. 3D lower limbs angles and moments were estimated, and total support moment (TSM) was calculated. Biomechanical outcomes were compared along the gait cycle (GC) (0%–100%) between groups using statistical parametric mapping (SPM).

**Results:**

The results showed higher KOOS total score for BUKS compared to OUKA (*p* = 0.020, effect size [ES] = 0.62). No significant differences were observed between BUKS and OUKA for the biomechanical variables (*p* > 0.05). Significant decrease of knee extension angle for OUKA compared to CG between 27% and 46% of GC (*p* < 0.001) was observed. Knee moments showed a significant decrease for the external knee flexor moment for OUKA compared to CG between 55% and 76% of the stance phase (SP) (*p* < 0.001). A reduction of the contribution of the knee to the first peak of TSM was observed for both BUKS and OUKA compared to CG (*p* = 0.019, ES = 0.34).

**Conclusions:**

BUKS patients demonstrated similar knee function compared to OUKA. OUKA group exhibited a protective mechanism by reducing the knee extension. Neither BUKS nor OUKA restored knee joint function comparable to a native knee, with compensation mechanism occurring through adjacent joints.

**Level of Evidence:**

Level III.

**Abbreviations**
ACL: anterior cruciate ligament
CG: control group
EMG: electromyography
GC: gait cycles
ISB: International Society of Biomechanics
KOOS: knee injury and osteoarthritis outcomes score
MCID: minimal clinically important difference
OA: knee osteoarthritis
PROMs: patient‐reported outcome measures
RMS: root mean square
SP: stance phase
SPM: statistical parametric mapping
TKA: total knee arthroplasty
TSM: total support moment
UKA: unicompartmental knee arthroplasty

## INTRODUCTION

In recent years, unicompartmental knee arthroplasty (UKA) has increasingly been considered as successful treatment for medial knee osteoarthritis (OA) [16]. Compared to total knee arthroplasty (TKA), UKA is characterised by its less invasive approach and, most importantly, by its preservation of the anterior cruciate ligament (ACL). Preserving the ACL contributes to superior knee function and high scores on patient‐reported outcome measures (PROMs) [24]. However, the revision rates remain higher for UKA [21]. The most common cause of revision includes disease progression to the contralateral compartment, aseptic loosening and bearing dislocation in mobile‐bearing designs [20]. Patient‐specific UKA was introduced as a solution for mismatched implanted sizing and has been used for over a decade, demonstrating a superior fit compared to off‐the‐shelf UKA [4]. Computational simulation using finite element analysis has also suggested that patient‐specific medial UKA may offer advantages by reducing contact stress on the lateral compartment, potentially helping to prevent the progression of tibio‐femoral knee OA compared to off‐the‐shelf designs [17]. However, the superiority of patient‐specific UKA over off‐the‐shelf UKA has not been proved yet. Considering the limited number of studies conducted and the necessity for longer follow‐up data, the current evidence on patient‐specific UKA remains inconsistent [9, 27]. Some studies have shown excellent improvements in the knee society score and high implant survivorship at 2‐year follow‐up, while others have reported a revision rate as high as 25% in a cohort of 115 implants using the same patient‐specific UKA design [11, 36]. From a functional perspective, a finite element study showed that patient‐specific UKA achieved closet kinematics in terms of tibial translation and rotation compared to native knee during gait and squat [22]. To our knowledge, no study has yet investigated the biomechanical outcomes of patient‐specific UKA [9]. Gait analysis has already provided objective metrics for assessing knee function following knee arthroplasty [5, 23]. A systematic review also demonstrated that UKA does not fully restore normal knee function similar to native knee during level walking [19]. Persistent abnormalities in individuals having UKA include slower gait velocity, reduced knee extension during mid‐stance [6, 40] and higher muscles activation around the knee [5].

Therefore, the aim of this study was to assess the potential differences between the personalised unicompartmental prosthesis (Bodycad UKS [BUKS]) and an off‐the‐shelf prosthesis (Oxford UKA [OUKA]) in knee joint function in terms of both biomechanical and clinical outcomes during level walking. The first hypothesis was that BUKS would provide superior knee function compared to OUKA, and the second hypothesis was that neither BUKS nor OUKA would fully restore normal knee function compared to native knee.

## MATERIALS AND METHODS

### Participants

In this study, 22 nonconsecutive medial Knee OA patients who underwent surgery between 2019 and 2022 were retrospectively recruited with the collaboration of an orthopaedic nurse of the CHU‐Québec.

Fourteen patients implanted with the Oxford mobile‐bearing UKA (Oxford Phase III Biomet UK Ltd.) and eight patients implanted with a BUKS, Fixed‐bearing UKA (Bodycad Inc.) were included. Patients were included if they met the following criteria: (1) aged between 45 and 75 years, (2) Kellgren and Lawrence grade 3 or 4 [18], (3) intact ACL, (4) minimum follow up period ≥ 18 months and (5) body mass index (BMI) ≤ 35 kg/m^2^. Exclusion criteria were: (1) contra‐lateral lower limb prosthesis, (2) pain in the contralateral knee and (3) musculoskeletal disease that may affect walking.

Twenty‐two healthy individuals matched for age and height were recruited for the control group (CG). Additional exclusion criteria for the CG were knee pain, lower limb arthroplasty or musculoskeletal diseases that may affect walking or balance. This study was approved by the local ethic committee of the CIUSSS de la Capitale‐Nationale (#2021‐2279) and participants signed the informed consent form. All participants demographic information is available in Table 1.

**Table 1:** Demographic information, follow‐up period and KOOS subscale values.

	BUKS	OUKA	CG	*p* value (ES)
Participants (female)	8 (6)	14 (7)	22 (13)	
Age (years)	70 ± 4	68 ± 5	68 ± 4	0.452
Height (m)	1.60 ± 0.09	1.67 ± 0.12	1.65 ± 0.10	0.291
Mass (kg)	82.9 ± 11.10	87.5 ± 20.45	67.2 ± 11.6	0.002^a^, ^b^
BMI (kg/m^2^)	32.6 ± 4.3	31.7 ± 6.2	24.6 ± 3.4	<0.001^a^, ^b^
Follow‐up (months)	54 (45–71)	39 (24–49)	N/A	<0.001^c^
KOOS—symptoms	92 ± 6	84 ± 12	N/A	0.178 (0.36)
KOOS—pain	92 ± 8	84 ± 19	N/A	0.336 (0.26)
KOOS—activities of daily living	93 ± 7	85 ± 19	N/A	0.631 (0.13)
KOOS—sports and recreation	77 ± 12	52 ± 27	N/A	0.043^c^ (0.54)
KOOS—quality of life	92 ± 15	76 ± 23	N/A	0.026^c^ (0.58)
KOOS—total	89 ± 7	76 ± 16	N/A	0.020^c^ (0.62)

Abbreviations: BMI, body mass index; BUKS, Bodycad unicompartmental knee system; CG, control group; ES, effect size using rank biserial correlation; KOOS, knee injury and osteoarthritis outcome score; OUKA, Oxford unicompartmental knee arthroplasty.

^a^ Significant difference between BUKS and CG.

^b^ Significant difference between OUKA and CG.

^c^ Significant difference between BUKS and OUKA.

### Surgery

Patient‐specific UKAs were manufactured based on MRI imaging of the affected knee. The patient‐specific instrumentation and implants were then manufactured after receiving the approval of the treating surgeons. A detailed explanation of the procedure has been previously published [3]. The knee was approached via a trans‐vastus parapatellar approach. Tibial and femoral implants were impacted, screwed in place and cemented, and the tibial insert thickness was chosen from a selection of inserts of millimetric increments based on the intraoperative knee ligamentous balance.

### Instrumentation and gait analysis

Gait analysis was conducted between April 2023 and April 2024 using a 10‐camera motion analysis system (Vicon, Vantage 5) and four force plates (AMTI®, OR6) embedded in 10‐m walkway. The acquisition frequencies for the motion analysis system and force plates were 100 and 1000 Hz, respectively. Participants were equipped with 32 reflective markers and eight clusters, creating a 6 degrees of freedom model for the lower limbs and pelvis, following the recommendation of the International Society of Biomechanics (ISB) [42], along with the Plug‐in‐Gait model for the trunk [39].

Muscle activations (electromyography [EMG]) were recorded using wireless sensors (Delsys Trigno) at a sampling frequency of 2000 Hz. A total of 14 sensors were placed bilaterally on lower limbs. Muscle activations were recorded for the Rectus Femoris, Vastus Medialis, Vastus Lateralis, Biceps Femoris, Semitendinosus, Tibialis Anterior and Gastrocnemius Medialis following the SENIAM's recommendations [14].

Participants were asked to walk barefoot at their self‐selected comfortable walking speed. Familiarisation trials were given at the beginning of the session to select the walking speed. Participants were asked then to maintain the same gait speed during all the trials. A trial was considered valid if the participant's foot fully touched the force plate. Additionally, the French version of the knee injury and osteoarthritis outcomes score (KOOS) was used [31].

### Data processing

Markers trajectories and ground reaction forces were filtered using a fourth order low‐pass Butterworth filter with a cut off frequency of 6 and 20 Hz, respectively. The Motion Monitor software (V3.74.0.0, Innovative Sports Training) was used to quantify kinematics, kinetics and EMG data. For the following analysis, only the operated lower limb was included for comparison for both UKA groups and right lower limb for CG.

Spatiotemporal parameters including gait speed, stride length, stride width and cadences were measured. Lower limb joints angles (hip, knee and ankle) were calculated using the Cardan–Euler sequence *XYZ* (*X* = Sagittal, *Y* = Frontal and *Z* = Transverse). External joint moments were estimated using Newton–Euler inverse dynamics equations and normalised to bodyweight. The average of 10 successful gait cycles (GC) were kept for kinematics analysis and time normalised to 101 data points from 0% (heel strike) to 100% GC (subsequent heel strike). Three successful cycles were selected for kinetics analysis and normalised to 100% stance phase (SP). The EMG data were filtered with a bandpass Butterworth filter with a cut‐off frequency of 20–450 Hz. For each muscle, the root mean square (RMS) was calculated using a 25 ms moving window then normalised using the peak RMS amplitude among all trails and time normalised to 101 data points from 0% to 100% GC.

All the normalisation procedures were conducted using BYK software (Moveck Solution Inc). The total support moment (TSM) was calculated using Winter's equation [41] by summing extensor moments of the hip, knee and ankle during the SP. The first and the second peaks were then extracted for comparison.

### Statistical analysis

*Participant's demographics, KOOS scores and spatiotemporal parameters:* The Shapiro–Wilk test was used to verify the normality of the distribution. In case of normally distributed data, parametric one‐way analysis of variance (ANOVA) was used to compare between three groups. For nonnormally distributed data, nonparametric ANOVA was applied. Tukey's post‐hoc was conducted to compare each group in case of significant main effect. the effect size (ES) was calculated with partial *η* ^2^ for all outcomes. All statistical analysis was conducted using Jamovi (Version 2.4.1.1) [37]. Significance's level was set at *p* ≤ 0.05.

*Kinematics, kinetics and EMG:* Joint kinematics, joint external moments, and EMG were analysed using statistical parametric mapping (SPM) (open‐source spm1D: www.spm1d.org) for full‐curve analysis. The normality of the data distribution was assessed using the function spm1d.stats.normality.anova. For normally distributed data, a one‐way ANOVA (SPM{*F*}) was performed, followed by two‐sample *t*‐tests (SPM{*t*}) for post hoc comparisons and Critical thresholds (t*) was reported. A Bonferroni correction was applied to the *t*‐tests and the *p*‐value was adjusted accordingly. In case of nonnormally distributed data, nonparametric tests (SnPM) were used. All analyses were implemented in a custom Matlab script (MATLAB R2024a, The MathWorks Inc.).

## RESULTS

The KOOS scores demonstrated a significant difference between both UKA design as BUKS showed higher total score compared to OUKA (89 ± 7, 76 ± 16, *p* = 0.020, ES = 0.62, respectively). All the subscales are presented in Table 1. Spatiotemporal parameters did not differ between BUKS and OUKA (*p* > 0.05, Table 1).

### Lower‐limb kinematics and kinetics

**Figure 1:**
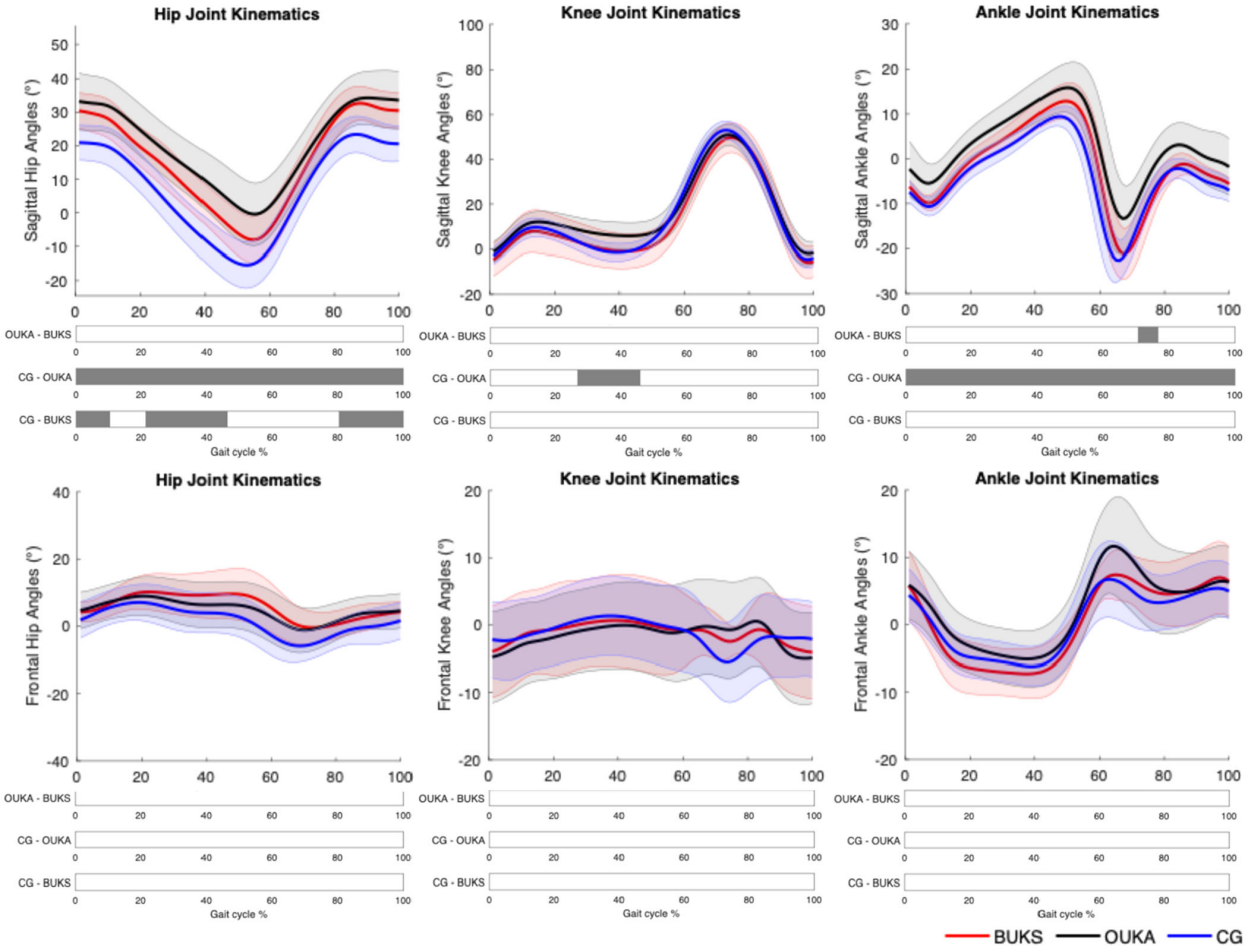
Lower limb sagittal and frontal plane kinematics during gait cycle. Significant differences between groups are indicated as a grey bar below the curves. BUKS, Bodycad unicompartmental knee system; CG, control group; OUKA, Oxford unicompartmental knee arthroplasty.

**Figure 2:**
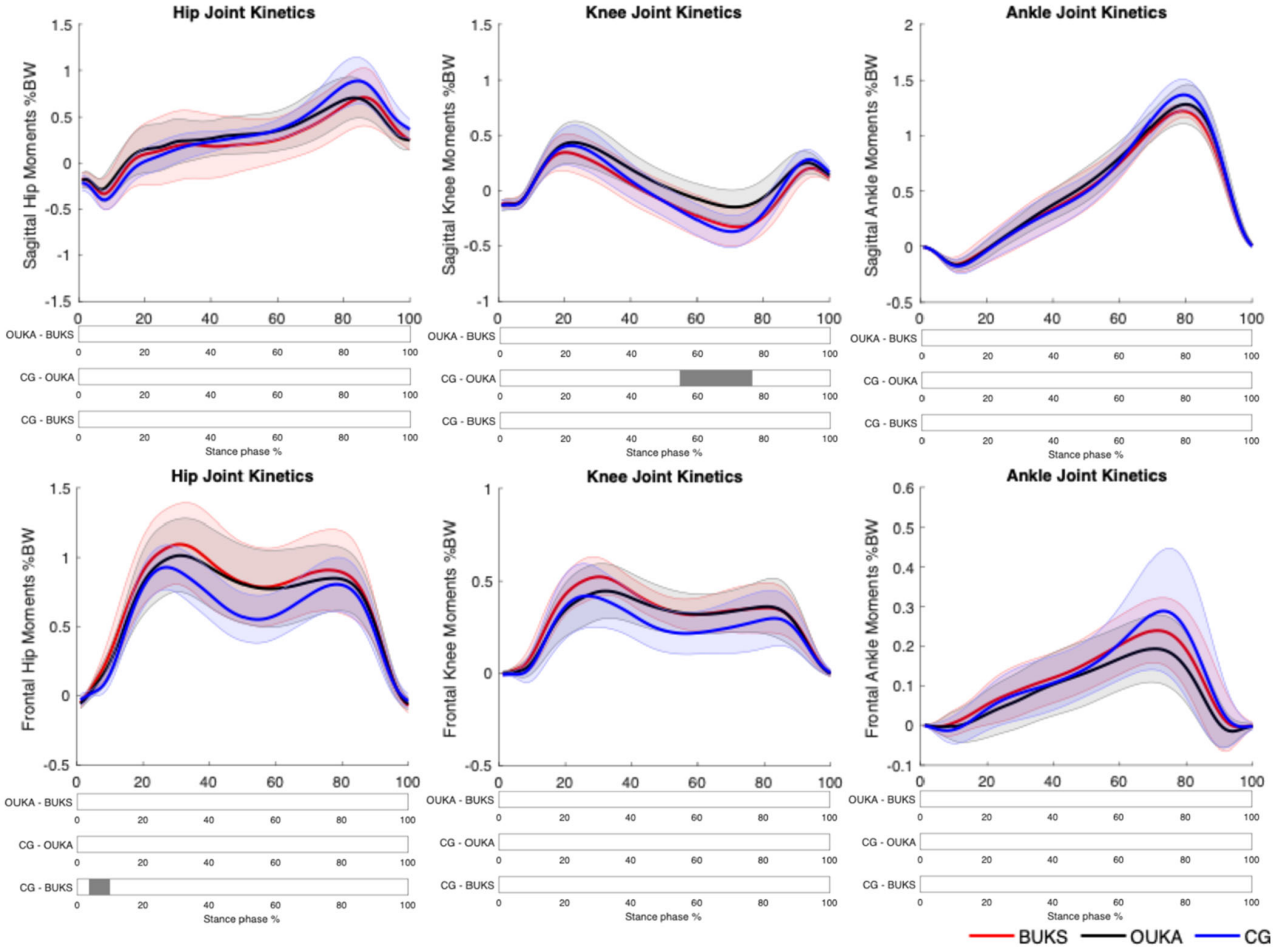
Lower limb sagittal and frontal plane kinetics during stance phase. Significant differences between groups are indicated as a grey bar below the curves. BUKS, Bodycad unicompartmental knee system; CG, control group; OUKA, Oxford unicompartmental knee arthroplasty.

The lower limb joint angles and external moments in sagittal and frontal plans are presented in Figures 1 and 2, respectively.

In sagittal plan, there were no significant differences for the hip and knee angles between BUKS and OUKA (*p* > 0.05). Meanwhile, BUKS showed an increase in hip flexion angle compared to CG during loading response, mid‐stance and late swing, between 0%–10%, 21%–46% and 80%–100% GC (t* = 3.42, *p* = 0.004, *p* = 0.003 and *p* = 0.002). OUKA group showed a similar significant hip flexion angle increase during the whole GC compared to CG (t* = 2.67, *p* < 0.001). At the knee, OUKA demonstrated a significant reduction in knee extension angle during mid‐stance, 27%–46% GC (t* = 3.46, *p* < 0.001).

At the ankle, OUKA group showed an increase in the dorsiflexion angles between 70%–77% GC compared to BUKS (t* = 3.15, *p* = 0.001) and during all GC compared to CG (t* = 2.74, *p* = 0.001). There were no significant differences between BUKS and OUKA in the frontal plan, nor compared to the CG. For net external moment, there were no significant differences between BUKS and OUKA (*p* < 0.05). OUKA showed a significant reduction of the external knee flexor moment during mid‐stance, 55%–76% SP (t* = 3.58, *p* < 0.001). BUKS showed a significant increase of the external hip abduction moment compared to CG during early stance, 3%–10% SP (t* = 3.57, *p* = 0.001). There were no significant differences for the remaining joints external moments.

### EMG

**Figure 3:**
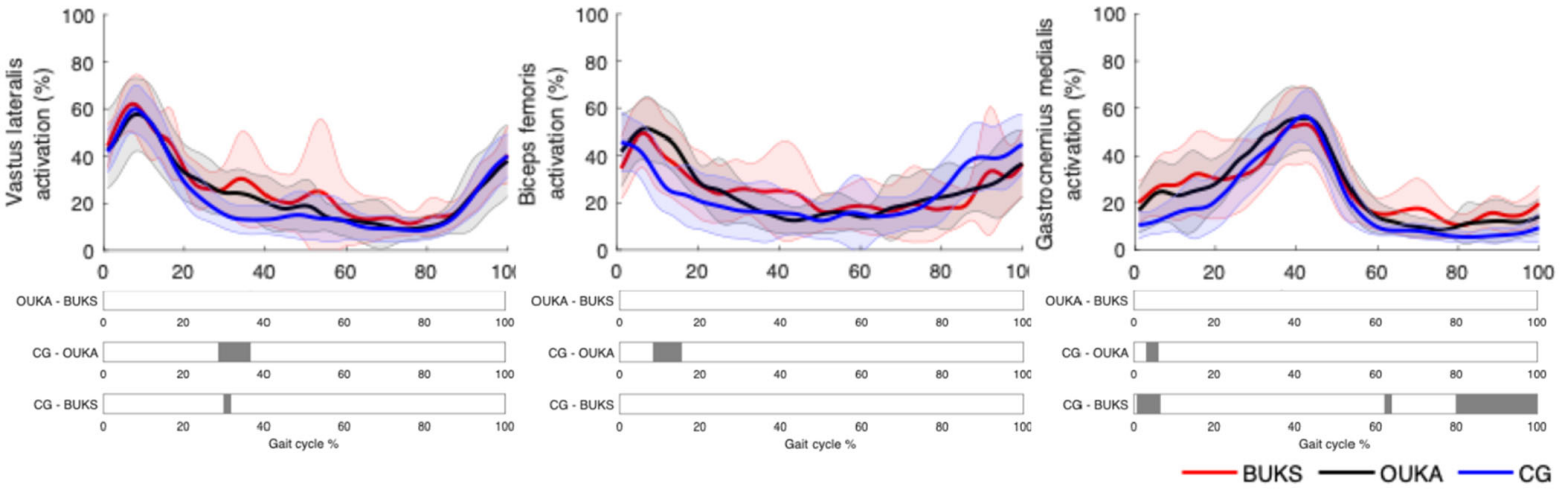
Muscle activation of the Vastus Lateralis, Biceps Femoris and Gastrocnemius Medialis during gait cycle. Significant difference between groups is indicated as a grey bar below the curves. BUKS, Bodycad unicompartmental knee system; CG, control group; OUKA, Oxford unicompartmental knee arthroplasty.

Only 13 out of 14 OUKA patients were equipped with EMG sensors due to a technical issue that occurred during data collection for one patient. All muscle activations are presented in Figure 3 and Supporting Information S1: Figure S1. There were no significant differences between BUKS and OUKA for the muscle activations (*p* > 0.05). Both OUKA and BUKS demonstrated an increase in *Vastus Lateralis* activation during mid‐stance compared to CG. While OUKA showed a significant increase between 29%–37% GC (t* = 3.44, *p* = 0.002), BUKS showed a similar increase in activation between 30%–32% GC (t* = 3.98, *p* = 0.006). OUKA elicited a significant increase in *Biceps Femoris* activation compared to CG during early stance, between 8%–15% GC (t* = 3.64, *p* = 0.001). Moreover, both BUKS and OUKA showed a significant prolonged activation of *Gastrocnemius Medialis* compared to CG. BUKS demonstrated a significant increase during early stance and late swing, 1%–7%, 62%–64% and 80%–100% GC (t*= 3.82, *p* = 0.001, *p* = 0.009 and *p* = 0.001). OUKA showed a significantly higher activation during the loading response, 3%–6% GC (t*= 3.60, *p* = 0.004).

### TSM

**Figure 4:**
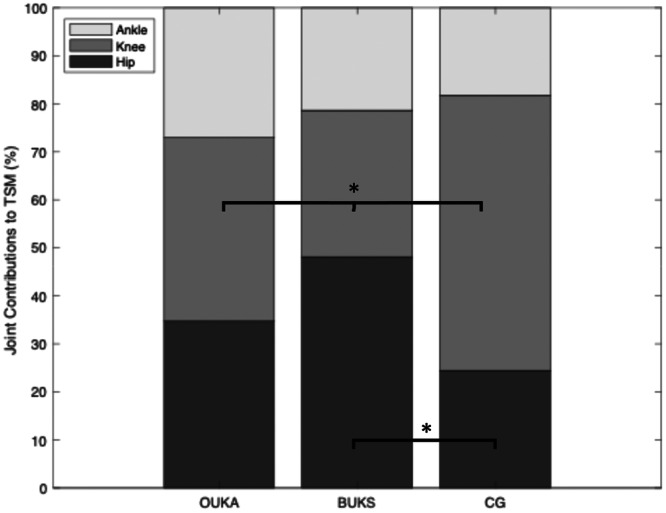
First peak TSM and joints contribution (*p < 0.05). BUKS, Bodycad unicompartmental knee system; CG, control group; OUKA, Oxford unicompartmental knee arthroplasty; TSM, total support moment.

The first and second TSM peaks and joints contributions are presented in Table 2. No significant differences were observed in the first peak of TSM between BUKS and OUKA, nor compared to the CG. The BUKS group showed an increased contribution of the hip to the first peak of TSM compared to the CG, 48% ± 20% (*p* = 0.035, ES = 0.64), Figure 4. Both BUKS and OUKA demonstrated a significant decrease in knee contribution to the first peak of TSM compared to CG, 30% ± 23% (*p* = 0.019, ES = 0.88), 38% ± 19% (*p* = 0.048, ES = 0.77), respectively. A significant difference was observed for second peak of TSM for OUKA compared to CG (*p* = 0.009, ES = 0.98). Specifically, OUKA showed a significant increase in the ankle contribution to the second peak of TSM compared to CG (*p* = 0.023, ES = 0.62).

**Table 2:** Spatiotemporal and TSM results.

	BUKS	OUKA	CG	*p* value (ES)
Spatiotemporal				
Gait speed (m/s)	1.09 ± 0.15	1.09 ± 0.20	1.23 ± 0.19	0.088 (0.21)
Stride length (m)	0.58 ± 0.08	0.59 ± 0.08	0.63 ± 0.08	0.167 (0.17)
Stride width (m)	0.09 ± 0.02	0.10 ± 0.03	0.08 ± 0.03	0.161 (0.16)
Cadence (step/m)	113 ± 8	112 ± 9	116 ± 10	0.506 (0.06)
TSM				
First peak TSM (Nm/kg)	0.63 ± 0.30	0.81 ± 0.33	0.61 ± 0.22	0.184 (0.18)
Hip contribution (%)	48 ± 20	35 ± 15	24 ± 21	0.012^a^ (021)
Knee contribution (%)	30 ± 23	38 ± 19	57 ± 25	0.019^a^, ^b^ (0.34)
Ankle contribution (%)	21 ± 19	27 ± 24	18 ± 16	0.738 (0.01)
Second peak TSM (Nm/kg)	1.94 ± 0.30	1.83 ± 0.37	2.19 ± 0.32	0.020^b^ (0.33)
Hip contribution (%)	28 ± 12	28 ± 10	35 ± 8	0.050^a^ (0.30)
Knee contribution (%)	9 ± 8	9 ± 4	9 ± 5	0.572 (0.03)
Ankle contribution (%)	63 ± 9	63 ± 9	55 ± 7	0.019^b^ (0.18)

Abbreviations: BUKS, Bodycad unicompartmental knee system; CG, control group; ES, effect size (*η*²); OUKA, Oxford unicompartmental knee arthroplasty; TSM, total support moment.

^a^ Significant difference between BUKS and CG.

^b^ Significant difference between OUKA and CG.

^c^ Significant difference between BUKS and OUKA.

## DISCUSSION

The objective of this study was to compare, from a biomechanical perspective, a personalised UKA versus an off‐the‐shelf mobile bearing UKA during level walking. To our knowledge, this is the first biomechanical study to investigate the performance of a personalised UKA design during level walking at self‐selected speed [10]. The main finding was that both UKA designs demonstrated good functional scores using the KOOS questionnaire and personalised UKA did not differ compared to off‐the‐shelf UKA from biomechanical perspective. However, higher muscle activation around the knee and a protective mechanics involving ipsilateral joints persists at a mid‐term follow‐up for both UKA designs.

Our findings are in accordance with the results of the systematic review and meta‐analysis conducted by Kim et al. [19], which suggest that both fixed and mobile‐bearing UKA designs fail to restore normal knee function comparable to native knee. Moreover, implant personalisation did not lead to full recovery of knee function.

Indeed, it was demonstrated that UKA patients exhibited a smaller knee extension angle during midstance compared to control suggesting that a lack of knee extension may be associated with impaired quadriceps function [19]. A quadriceps dysfunction was already estimated to be close to 30% compared to healthy individuals at 2 years follow‐up [12]. Our results align with this hypothesis, showing higher activation of the *vastus lateralis* for both prosthesis design during mid‐stance and an increase of *biceps femoris* activation for OUKA patients compared to control during early stance. In this study, OUKA patients exhibited similar reduction of knee extension angles and smaller external knee flexor moment during midstance compared to CG. One should note that the follow‐up period of included studies in this systematic review is shorter (11.8–22.1 months) than the present study (39 months for OUKA group), which may suggest that these abnormalities can persists even at mid‐term follow‐up.

Fewer studies have investigated muscle activation during level waking for UKA [5, 13]. Indeed, Catani et al. [5] demonstrated abnormal muscle activation around the knee in two different UKA designs (i.e., fixed and mobile bearing). In their study, only the activation of the rectus femoris was recorded from the quadriceps muscle group, which may limit direct comparisons. Additionally, a limitation to consider is that the CG consisted of young adults, whereas the prosthesis group included older individuals.

A majority of the biomechanical studies conducted on UKA focus only on the knee [1], and does not take into consideration any information about ipsilateral joints (ankle and hip) [5, 26, 28, 30]. However, one of the main causes of UKA revision is the progression of OA to the lateral compartment or contralateral knee [20], which may lead to compensatory mechanics to reduce pain similar to the preoperative state [43]. In previous studies [29, 41, 43], the TSM was employed to provide insight concerning the contribution of each joint to the TSM during the SP. Hence, it could represent a relevant, reliable and less variable measurement of lower limb joint compared to separated joint external moment [41]. In the present study, the first peak of TSM did not differ between groups, however, the contribution of each joint changed. BUKS and OUKA patients tended to reduce the knee's contribution and compensate more with the hip. These results suggest that even at 5 years follow‐up patients may still exhibit preoperatively protective mechanisms and have less confidence putting more loads on the operated knee [1]. The advantage of a personalised UKA design compared to an off‐the‐shelf UKA design remains unclear [9, 27]. Better PROM results have been reported for personalised UKA design [2, 11, 34]. In this study, the BUKS group demonstrated a higher KOOS score compared to the OUKA group, with particularly notable improvements in the Sports and Recreation and Quality of Life subscales. The differences observed in these subscales exceeded the established minimal clinically important difference (MCID) thresholds, indicating a clinically meaningful improvement in patient‐reported outcomes [25]. However, these PROM results should be interpreted with caution, as both investigators and BUKS patients were unblinded due to patients' consent to receive a personalised implant. Moreover, while the KOOS is a primary designed to assess symptoms, pain and functional limitations, it's sensitivity to detect specific biomechanical changes is limited [32]. A recent study [7] suggested that the KOOS may not be suitable for detecting changes in the knee joint mechanics, and it is therefore recommended to combine PROM's with objectives metrics such as kinematics, kinetics and EMG outcomes.

From a biomechanical perspective, the personalisation of UKA did not lead to a superior knee function compared to off‐the‐shelf UKA. However, a tendency towards a better knee extension during mid‐stance was observed (Figure 1), but the sample size of this personalised UKA cohort was small to confirm this finding. The patient‐specific implant investigated in this study was a fixed‐bearing design (BUKS) and compared to a mobile‐bearing (Oxford Phase III). In the literature, there was no evidence regarding the superiority of one design compared to the other, as both designs had comparable revision rates [33] and functional outcomes [5]. In addition, neither BUKS nor OUKA demonstrated knee function similar to the CG at a mid‐term follow‐up, and some abnormalities persisted, including higher muscle activation around the knee and compensatory mechanisms involving ipsilateral joints.

Some limitations should be noted. First, the results of personalised UKA should be interpreted with caution due to the small sample size of the personalised UKA group. A priori sample size analysis indicated that a minimum of 18 participants per group was required to achieve adequate statistical power (80%). However, due to limitations in the availability of patients who received the personalised implant, it was not possible to reach this target sample size [15]. Similar biomechanical studies on UKA also included smaller sample size [8, 35, 38], primarily due to restrictive inclusion criteria related to the progression of OA or arthroplasty in the contralateral limb. Second, PROM results may be influenced as the investigators and BUKS patients were unblind during this study. Third, the arthroplasty groups had a higher BMI than the CG, which may impact gait analysis procedures and EMG outcomes. However, we normalised external joint moment for bodyweight to minimise any potential impact of body weight on our external joint moment. Further randomised studies with higher sample size are needed to better understand the effects of UKA personalisation.

## CONCLUSION

At mid‐term follow‐up, BUKS patients demonstrated superior clinical scores but failed to exhibit superior knee function, specifically in terms of knee extension during mid‐stance and muscle activation compared to OUKA. The OUKA group exhibited a protective mechanism by reducing the knee extension during SP, similar to what is observed preoperatively with OA patients. Neither BUKS nor OUKA restored knee joint function comparable to a native knee, with compensation mechanism occurring through adjacent joints. Further analyses are required to validate these findings in other, more challenging functional tasks such as squat, stair negotiation and sit to stand.

## AUTHORS CONTRIBUTIONS

**Haithem M'barki**: Conceptualization; methodology; formal analysis and investigation; writing—original draft preparation. **Etienne L. Belzile**: Conceptualization; methodology; writing—review and editing; funding acquisition; resources; supervision. **Katia Turcot**: Conceptualization; methodology; writing—review and editing; funding acquisition; resources; supervision.

## CONFLICT OF INTEREST STATEMENT

Etienne L. Belzile is Associate editor for Orthopaedics & Traumatology; Surgery & Research, and received research support from CIHR, AOSSM, Arthritis Society and MITACS, and is a consultant for Pendopharm and BodyCad. The remaining authors declare no conflicts of interest.

## ETHICS STATEMENT

This study was approved by the local ethic committee of CIUSSS de la Capitale‐Nationale (#2021‐2279). A written informed consent was obtained from participants to participate in the study.

## Supporting information

Supporting Information.

## Data Availability

The data that support the findings of this study are not publicly available due to privacy reasons but are available from the corresponding author upon reasonable request.
