# Supplementary material for: Patient‐specific versus off‐the‐shelf unicompartmental knee arthroplasty during level walking
Source: J Exp Orthop. 2025 Aug 27;12(3):e70347. doi: 10.1002/jeo2.70347 (PMC12381529; doi:10.1002/jeo2.70347)
Supplement: Supplementary file 1 — Supporting Information. [file JEO2-12-e70347-s001.docx]

Appendix

Supplementary Figure 1: Muscle activation of the Rectus Femoris, Vastus Medialis, Semitendinosus and Tibialis Anterior during gait cycle. Significant difference between groups is indicated as a gray bar below the curves.
